# Spotted Child

**Published:** 1853-10

**Authors:** 


*Spotted Child.*—The Boston Medical and Surgical Journal learns from the Manchester, N. H., Mirror, that a spotted child was recently born at Barnstead. "One half of the head, including one half of the forehead, is black, while the counter half is white. The face below the eye-brows, assumes an ash-yellow." The other parts of the body, except the shoulders, which are spotted, are white.

